# Transness is our salve: How trans identity facilitates healing from relational trauma with parental figures

**DOI:** 10.1002/jts.70044

**Published:** 2026-01-28

**Authors:** Joonwoo Lee, Sebastian Barr, Alberta M. Gloria, Tonya J. Roberts, Stephanie L. Budge

**Affiliations:** ^1^ Department of Counseling Psychology University of Wisconsin–Madison Madison Wisconsin USA; ^2^ Independent Practice Northhampton Massachusetts USA; ^3^ School of Nursing University of Wisconsin–Madison Madison Wisconsin USA

## Abstract

Transgender and nonbinary (TNB) individuals experience high rates of relational trauma from parental figures, yet their pathways to healing remain underexplored. This qualitative study used constructivist grounded theory to develop a theoretical framework of how TNB adults heal from parental relational trauma. In‐depth interviews with 15 racially and socioeconomically diverse U.S.‐based TNB adults who experienced parental relational trauma generated the core phenomenon: “TNB identity and experiences initiate and sustain pathways of healing from parental relational trauma.” This captures that rather than being a source of this trauma, recognizing and actualizing one's TNB identity is the central catalyst for healing from it. The healing process began with three conditions: (a) connecting with one's TNB identity/self, (b) gaining new clarity in parental harm through TNB‐related parental harm, and (c) experiencing joy and oppression as TNB while lacking parental support. These conditions gave rise to strategies including (d) creating safer homes through queer/TNB chosen family, (e) committing to individual healing through therapy, and (f) reimagining life through ongoing actualization as TNB. The consequences of these strategies, in turn, created conditions that enabled subsequent strategies, including (g) attempting repair and intergenerational work with families of origin, (h) distancing as self‐preservation, and (i) returning to TNB communities to sustain healing across generations. The consequences of these processes included personal, intergenerational, and collective healing. The study offers implications for research, clinical practice, and advocacy, calling for approaches that frame TNB identity not as a source of familial conflict but as a central resource for recovery.

Relational trauma, also known as complex or developmental trauma, is characterized by patterns of chronic abuse, neglect, or rejection that occur within relationships that are expected to offer attunement and safety (Courtois &Ford, 2012; Herman, 2015; Schore, 2001). This type of trauma differs from acute stressors (e.g., natural disasters) or trauma inflicted by strangers and has been shown to have particularly lasting effects on emotion regulation, self‐concept, interpersonal capacities, and resilience (Fares‐Otero et al., 2025, 2023). The 2015 U.S. Transgender Survey found that more than one in four transgender and nonbinary (TNB) individuals who were “out” (i.e., had disclosed their gender identity) to their immediate family reported experiencing estrangement (James et al., 2016), and TNB adults report higher rates of relational trauma involving parental figures (Thoma et al., 2021). Despite this, the experiences and healing trajectories of TNB survivors of relational trauma remain underexplored.

## Review of the literature

A growing body of research on the general population identifies key factors in healing from relational trauma involving parental figures. A meta‐analysis of adult survivors highlighted intrapersonal (e.g., self‐concept, emotion regulation, coping skills) and interpersonal factors (e.g., authenticity in relationships, lower perceived burdensomeness) that mediate psychological resilience after trauma (Fares‐Otero et al., 2025). Qualitative studies similarly emphasize intrapersonal shifts—such as reclaiming autonomy, making meaning of trauma, and developing emotional attunement (Stige et al., 2013)—and interpersonal changes, including breaking harmful patterns, setting boundaries, and engaging in mutual care (McCormack & Katalinic, 2016), as sources of healing. In addition, establishing relational safety, defined as a felt sense of affirming, attuned, and well‐boundaried connection that does not reproduce prior harm, within therapeutic and peer contexts further supports recovery (Grossman et al., 2017). Contextualizing relational trauma within intergenerational and historical oppression can also deepen healing (Payne et al., 2013), as can sharing one's story and engaging in service for others with similar experiences (Gwozdziewycz & Mehl‐Madrona, 2013). These findings align with trauma treatment models emphasizing emotional regulation, relational safety, and narrative integration as core sources of healing from relational trauma with parental figures (Grossman et al., 2017; Herman, 2015).

However, the experience of parent‐induced relational trauma and the associated healing processes may differ significantly for TNB survivors. The trauma itself may stem directly from the person's gender identity (Singh & McKleroy, 2011) or be compounded by minority stress across socioecological levels, as TNB people face pervasive interpersonal invalidation and systemic oppression, both of which are chronic sources of trauma (Barr et al., 2022). Pathways to recovery accessible to cisgender survivors are often obstructed; for instance, although psychotherapy is a primary modality for trauma recovery, many TNB individuals face barriers, including provider incompetence, discrimination, and structural barriers (e.g., insurance coverage; Compton & Morgan, 2022).

Simultaneously, TNB people demonstrate an incredible capacity for healing, generativity, and resilience. Among TNB people of color, resilience following traumatic events is grounded in pride in one's gender and ethnic/racial identities; recognition of and resistance to oppression; connection with activist TNB communities of color; spirituality; hope; and, when accessible, support from family of origin, transition‐related health care, and financial stability (Singh & McKleroy, 2011). Broader research on TNB resilience has identified similar protective factors, including belonging, self‐definition, self‐worth, and serving as role models within one's communities (Matsuno & Israel, 2018).

Given these distinct challenges and strengths, the healing processes for TNB survivors may differ from those in cisgender populations. Emerging clinical literature has begun adapting trauma‐informed care to the unique needs of trans communities. Scholars emphasize affirmation and relational attunement to foster safety and stabilization in early treatment phases (Barr et al., 2024; Ellis, 2020; Minshew, 2022). Nascent research on trans‐affirming narrative exposure therapy that integrates experiences of nonaffirmation and transnegative bias has also shown promise as process‐phase interventions (Julian et al., 2024; Lange, 2020). However, this research remains limited. The present study addresses this gap by exploring the lived experiences of healing among TNB adults who have experienced relational trauma with parental figures, using constructivist grounded theory (CGT; Charmaz, 2014) to delineate key conditions, strategies, consequences, and variations of the healing process, providing a foundation for clinical practice, future research, and advocacy.

## METHOD

### Participants

TNB adults (*N* = 15, *M*_age_ = 32.93 years, *SD =* 12.29, range: 18–68) with diverse racial, ethnic, and socioeconomic backgrounds participated in the study. Participants identified as trans men/transmasculine (*n* = 4), trans women/transfeminine (*n* = 4), or nonbinary/gender expansive (*n* = 7). Participants identified as White (*n* = 9), multiracial (*n* = 5), Black/African American (*n* = 4), Asian American (*n* = 4), Indigenous/Native American (*n* = 3), and Latine (*n* = 2). Because participants were counted across all categories with which they self‐identified—including each category selected by multiracial participants—these categories are not mutually exclusive, and the White category includes individuals who also identified with other racial/ethnic groups. Regarding socioeconomic background, participants reported being working class (*n* = 8), working‐middle class (*n* = 3), middle class (*n* = 2), and upper‐middle class (*n* = 2). Participants reported having a bachelor's degree (*n* = 6), a high school diploma or GED (*n* = 3), some college experience (*n* = 4), or a graduate or professional degree (*n* = 2).

### Procedure

This study employed constructivist grounded theory methodology, a qualitative methodology in which theory is inductively generated from the data and coconstructed by researchers and participants. CGT is particularly well‐suited for examining underexplored and marginalized experiences, as it allows theory to emerge directly from participants’ lived realities and remain responsive to their sociocultural contexts.

#### Recruitment and data collection

This study was approved by the Institutional Review Board at the University of Wisconsin‐Madison. A two‐phase sampling process consistent with CGT (Charmaz, 2014) was used, beginning with purposive sampling and transitioning to theoretical sampling. Eligibility criteria included being at least 18 years old, identifying as TNB, and being able to communicate in English. Participants were also required to have a history of relational trauma with parental figures, defined as repeated emotional or physical abuse or neglect resulting in ongoing psychosocial effects.

##### Phase 1: Purposive sampling and initial screening

Participants were initially recruited via flyers on TNB social media pages. An online screening survey collected demographic data, confirmed eligibility, and included an open‐ended item inviting participants to describe their experiences of relational trauma. This yielded 284 responses. After removing suspected bot entries identified by duplicate, inconsistent, and/or suspicious data, 143 eligible individuals remained. From this pool, seven participants were selected for the first round of interviews (purposive sampling) based on their open‐ended screening responses. Participants were purposively selected to maximize variation in relational trauma experiences (e.g., emotional and physical abuse and neglect) and to prioritize individuals holding multiple marginalized identities (e.g., across race, socioeconomic status, and disability) in support of intersectional representation.

##### Phase 2: Theoretical sampling

To recruit participants who could clarify, expand, or challenge our developing categories, we distributed a second screening survey. A total of 80 individuals from the initial pool completed this survey, and eight participants were selected for additional interviews. Using a survey that combined forced‐choice items assessing key dimensions of healing with open‐ended items inviting elaboration, we intentionally recruited participants who reported high levels of healing from relational trauma through sources of healing identified in earlier purposive sampling (e.g., therapy, community connection, distancing, and repair attempts with parental figures).

#### Interviews

Data were collected through in‐depth, semistructured interviews that lasted up to 2 hr hours and were conducted using a HIPAA‐compliant video platform (Lyssn). The first author conducted 12 interviews, and the second author conducted three interviews. Interviews were audio‐recorded and transcribed verbatim. Consistent with CGT, the interview protocol was flexible and evolved iteratively. Phase 1 interviews explored broad experiences of trauma and healing, whereas Phase 2 interviews focused on conditions and processes of healing that had emerged as theoretically significant (see Supplementary Materials for the full protocol).

Following trauma‐informed qualitative principles (Isobel, 2021), interviews emphasized safety, choice, and reciprocity. Participants provided informed consent, could skip questions or withdraw, and set their own pace of disclosure. The interview protocol balanced an exploration of traumatic experiences with reflections on strengths and trans joy. Each session ended with a debrief offering psychoeducation and resources, positioning interviews as spaces of validation and care. The research culminated in a collaborative “zine” (i.e., a magazine‐style publication) cocreated with participants to return knowledge to the community.

#### Researcher positionality

This study was designed and analyzed by two transgender men whose personal and professional experiences informed the research. The first author, a South Korean transgender man, brought lived experience of the research topic as well as clinical and research experience in TNB trauma recovery. His community engagement, clinical practice, and training in CGT informed theoretical sensitivity and methodological decisions. The second author is a White transgender man and practicing counseling psychologist specializing in posttraumatic stress disorder (PTSD) and complex trauma. He has received advanced pre‐ and postdoctoral training in complex traumatic stress and has authored numerous publications on trauma‐informed care in TNB populations. The authors’ combined lived experience in the TNB community and professional expertise enhanced rapport with participants and supported nuanced analysis. The remaining coauthors, who also served on the first author's dissertation committee, provided expert review in intersectionality, CGT, manuscript preparation, and project management. They contributed diverse disciplinary backgrounds (counseling psychology and nursing) and social identities (people of color and White, cisgender, queer, and straight), enriching reflexivity and interpretive depth.

### Data analysis

Using ATLAS.ti (Lumivero, n.d.),a qualitative coding software, we conducted iterative cycles of open and focused coding, guided by constant comparison and reflexive memoing. During open coding, two coders independently conducted a line‐by‐line analysis to identify significant actions and meanings. Weekly meetings were held to compare and refine interpretations. Focused coding occurred concurrently, during which open codes were grouped into higher‐level categories, and their relationships were diagrammed to clarify emerging patterns and guide further theoretical refinement and data collection. This iterative process of moving between data collection and analysis continued until theoretical sufficiency was achieved—that is, when categories were well developed, and their relationships coherently supported the emerging theory (Dey, 1999). To ensure trustworthiness, we incorporated multiple strategies throughout the research process. Reflexivity was central, with coders engaging in ongoing reflective dialogue to ensure their positionalities enriched rather than biased the analysis. In weekly collaborative coding meetings, both researchers compared their independently coded interpretations through reflexive dialogue. A comprehensive audit trail documenting analytic decisions was maintained in ATLAS.ti and later reviewed by an external auditor.

Two external auditors provided additional credibility checks. The first auditor, a cisgender Chicana who identifies as having a disability and focuses on intersectional and qualitative research, reviewed our codebook and analytic structure, offering guidance that deepened the intersectional perspectives within our theory. The second auditor, a Black, African American, queer, spiritual, transmasculine early‐career academic from the southern United States, reviewed our theoretical framework and guided us in strengthening antiracist praxis and language, particularly in how we contextualized the experiences of participants of color relative to white participants.

Member‐checking was conducted with six participants through 45‐min, one‐on‐one videoconference meetings following the completion of coding. Participants offered rich insights, many affirming the framework's strong resonance with their lived experiences. Several individuals shared that the theory articulated healing processes they had previously experienced but struggled to express, whereas others noted that it helped them locate themselves within different stages of recovery. Participants also identified nuances and discrepancies, which directly informed refinements to the final theoretical model.

To support transferability, we provide detailed descriptions of participant demographics, social contexts, and our recruitment and analytic processes, allowing readers to assess the applicability of our findings to other contexts. Our purposive sampling strategy emphasized diversity across gender identities, racial and ethnic backgrounds, and geographic regions to ensure the resulting theory reflects the breadth of TNB lived experiences.

## RESULTS

### Core phenomenon: TNB identity and experiences initiate and sustain pathways of healing from parental relational trauma

**FIGURE 1:**
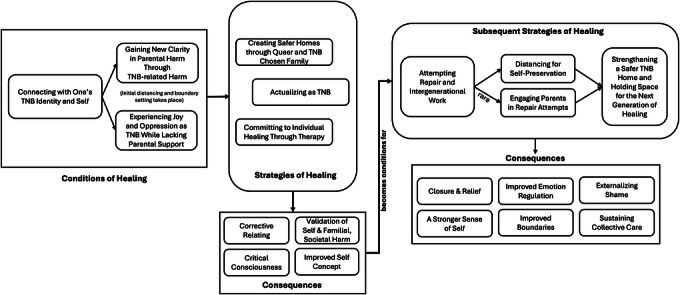
Diagram on the process of healing from parental relational trauma among trans and nonbinary (TNB) people

Our analysis generated a grounded theory describing how TNB adults heal from relational trauma involving parental figures. The theory centers on the ways TNB identity and experiences both initiate and sustain recovery processes. The process began with three foundational conditions: (a) connecting with one's TNB identity and self, (b) gaining new clarity in parental harm through TNB‐related parental harm, and (c) experiencing joy and oppression as TNB while lacking parental support. Building on these conditions, participants engaged in three core healing strategies: (a) creating safer homes through queer and TNB chosen family, (b) committing to individual healing through therapy, and (c) actualizing as TNB. The consequences of these strategies then prompted subsequent healing strategies to develop: (d) attempting repair and intergenerational work with families of origin, (e) distancing for self‐preservation, and (f) returning to TNB communities to sustain healing across generations. The consequences of these processes included personal, intergenerational, and collective healing. These processes are represented in the theoretical model shown in Figure 1. Participants selected their own pseudonyms, which are used throughout the findings.

### Condition for Healing 1: Connecting with one's TNB identity and self

Recognizing and connecting with TNB identity emerged as a foundational condition for healing from relational trauma with parental figures. As participants connected with their authentic gender, they described increased attunement to their emotional and bodily needs—ones that many had struggled to access due to their parents’ lack of ability to attune to participants’ emotions and discouragement from developing a clear sense of self. This awareness contrasted sharply with earlier dissociation (i.e., feeling disconnected from one's emotions or body) and fostered a desire for self‐care. One participant, Fly, described this shift in the following way: “I realized I live in my body, and I can't just throw it around anymore. … I was in my body and taking care of myself in a different way.” Turning toward internal truth also enabled participants to cultivate values distinct from those of their family of origin, creating distance from restrictive familial narratives. Forest, another participant, explained, “Developing my queer and trans identity helped a lot in that [self‐concept] because I was kind of going against what I was told.” Such attunement to one's emotions, needs, and values became an important condition for later strategies of healing.

Familial and systemic factors shaped access to this condition. Trans women often described delays in accessing this condition due to cisnormative and transmisogynistic familial and societal expectations and control imposed in response to their early gender diverse desires and behaviors. Some TNB participants of color noted barriers due to limited trans visibility within their racial communities, cultural norms opposing gender nonconformity, or limited material resources for gender transition.

### Condition for Healing 2: Gaining new clarity in parental harm through TNB‐related parental harm

As participants connected with TNB identities, expressions of gender nonconformity or coming out (i.e., disclosing one's gender identity) to parental figures often resulted in preexisting patterns of parental abuse being redirected toward the participant's gender identities. For example, longstanding patterns of shaming or belittling were channeled into transphobia, whereas prior neglect extended into refusal to support TNB‐related needs. Although this redirection of harm toward TNB identity triggered profound betrayal, it also brought unexpected clarity. For many participants who had struggled to acknowledge parental harm growing up, seeing it now directed toward their gender identity became a defining moment in recognizing longstanding relational harm. One participant, Fierce, described how their parent's neglect in TNB‐specific contexts illuminated longstanding parental neglect:

I realized that standing in my own authenticity and essentially raising myself was just the way it was going to have to be. … I began the process of realizing that I would not be able to depend on my parent for anything…that she was not going to get any better at this.

Recognizing this continuity and redirection of harm became a critical condition shaping subsequent healing strategies.

### Condition for Healing 3: Experiencing joy and oppression as TNB while lacking parental support

Living authentically as TNB brought participants both joy and oppression. This dual experience became an important condition of healing by exposing the limitations of existing relationships—particularly with parental figures—to hold the complexity of their lives. In many cases, parental support was withdrawn during gender transition, compounding the effects of parental and societal rejection. These losses prompted participants to seek new sources of social support. At the same time, moments of affirmation and embodied joy intensified their desire to belong in spaces where their authenticity and joy could be fully celebrated. Together, these contrasting experiences—joy and rejection—highlighted the necessity of cultivating new relational networks beyond the family of origin and engaging in intentional healing. In this way, the combined weight of relational trauma and societal oppression became conditions for strategies of healing.

### Strategies for Healing 1: Creating safer homes through queer and TNB chosen family

As participants developed greater self‐attunement and clarity of self (Condition 1), gained insight into parental harm (Condition 2), and recognized the need for new sources of support amid the dual experiences of TNB joy and oppression (Condition 3), they engaged several core strategies of healing. One key strategy was creating chosen families within queer and TNB communities.

Participants sought connection in queer and TNB spaces where shared experiences of marginalization fostered unique relational safety. These individuals trusted that others with shared experiences of familial and societal rejection would understand, validate, and respect their boundaries around their pain. One participant, Aphrodite, explained, “When I'm interacting with trans and queer people…it comes with a level of trust these people have also felt this pain. They will be likely to at least try to not hurt me in this way.” Beyond shared pain, these spaces were where participants could fully celebrate their transness. Another participant, Thunder, emphasized intentionally choosing such spaces to “embrace trans joy” instead of constantly “fighting for myself.”

Participants’ social locations influenced their access to this relational safety, which they most often found within communities that reflected their intersecting identities, such as race and socioeconomic status. For TNB participants of color, healing was most often found in communities created by and for queer and TNB people of color. Participants noted that White supremacy and White‐centric gender norms within broader queer and TNB spaces often reproduced harm. One participant, BrooklynA, reflected:

I don't think that White people have experienced the level of pain that Black and Brown folks have, and I don't think they're even capable of giving that [healing] to themselves. So how can I expect them to give it to me? We know what it's like to have our parents dismiss us, to have our families dismiss us, and then ultimately have White supremacy be replicated in these communities that we live in. So, there's nothing more powerful than us healing each other.

These intersectional spaces were not always accessible, as they were more common in large cities and could be complicated by intracommunity racial or ethnic differences. Still, participants often relocated or actively built community to create these safer spaces.

From connecting with queer and TNB chosen family, participants experienced (a) healthier and corrective relating and (b) validation of self and familial and societal harm.

#### Experiencing corrective relating

Forming queer and TNB chosen families provided corrective experiences of care. Participants described unconditional acceptance as a community ground rule that contrasted with the conditional love of their families of origin, helping them redefine what they deserved in relationships. One participant, Noah, shared:

My transness has endowed me with a community of people who love me for my proliferations, for my abundance. … I [realized], “Oh my gosh, maybe this is what I deserve.” They introduced me to something beyond what I was taught that I should just accept…and they showed me what love in action actually looks like.

Chosen families also served as spaces to relearn healthy boundaries and conflict repair; relational skills were rarely modeled in participants’ families of origin. Experiencing that ruptures could be repaired with care was profoundly healing. BrooklynA shared:

With my chosen family, when we would have arguments…there was none of that [violence]. We addressed the problem as soon as it happened. There was healing. It was love, it was kindness, it was affection. It was transformative.

#### Validation of the self, and parental and societal harm

Being validated and mirrored by others with shared experiences helped participants develop clearer, more positive self‐concepts while reducing internalized transphobia. One participant, Red, shared, “Realizing a sense of self by finding other people like me at the time. [Before,] I had no sense of self.”

In addition, witnessing others navigate similar parental and societal harm provided validation, contextualization, and clarity about participants’ own experiences. Through these shared reflections, participants came to situate their personal histories within systemic structures of gender, race, and intergenerational trauma. Noah emphasized how collective dialogue within their “queer commune” created space to critically unpack colonial histories shaping both parental and societal harm. Together, these narratives illustrate how chosen communities not only validated participants’ pain but also deepened their awareness of the broader historical and structural forces underpinning it.

Whereas most participants described chosen families as central sources of healing, some acknowledged that unresolved trauma could surface within these relationships. Red reflected that such moments highlighted the importance of pairing collective healing with individual healing. They noted that engaging in individual healing supported both their personal growth and their capacity to engage more positively within chosen family.

### Strategies for Healing 2: Committing to individual healing through therapy

Alongside collective healing in chosen families, participants engaged in the strategy of individual healing through therapy. The combined weight of relational trauma and societal oppression, reinforced by TNB community values emphasizing healing, motivated many participants to seek professional support, often as the first in their families to do so.

Therapy offered a space to process parental trauma and its ongoing effects, develop a clearer understanding of its impact, and learn concrete coping skills such as emotional regulation and boundary setting. It also provided long‐denied validation of abuse, helping participants externalize shame. One participant, Echo, reflected,

Going to therapy, and having another person validate those experiences and my emotions, made it a lot easier to know that I wasn't crazy, that I wasn't being difficult…that the things I experienced were abuse…and that it was not okay.

Accessing therapy was not always straightforward. Familial and cultural taboos around mental health created barriers, requiring some participants to first confront internalized stigma. As BrooklynA noted, they had to “decolonize” their own hesitations rooted in community norms. Others described challenges with providers who lacked cultural understanding and awareness of historical and contextual considerations. Fly emphasized the importance of therapists who could situate family trauma within broader historical forces, such as war and displacement, rather than reducing it to individual pathology. Despite these challenges, therapy emerged as a crucial strategy of healing—a space where participants could make sense of their parental trauma, receive affirmation, and develop practical tools for recovery.

### Strategies for healing 3: Actualizing as TNB

Whereas connecting with TNB identity was a condition of healing, the fuller strategy of *actualizing* the identity became a key strategy wherein participants reclaimed the self, actualized their potential, and developed critical consciousness of parental and societal harm.

#### Reclaiming the self and actualizing potential

Actualizing one's TNB identities enabled participants to reimagine life beyond restrictive family norms and reconstruct a sense of self grounded in internal truth rather than external expectations. Learning to affirm their TNB identity in the face of societal invalidation strengthened participants’ capacity to self‐validate, including the parental harm they had endured. Red reflected, “If I assert myself as a person by being trans, then I have to assert that I was hurt as a child.” This process also opened pathways for exploring other dimensions of identity, such as cultural, racial, and ethnic heritage, contributing to a more integrated self‐concept. For one participant, Sage, gender transition also facilitated a deeper connection to their Indigenous identity—a dimension of self they had previously been unable to connect amid difficult relationships with parental figures. Sage shared, “The introspection of trying to understand my gender and sexuality has made me very curious about my heritage.”

As participants deepened their connection to their internal truth and engaged in experiences rooted in authentic joy, rather than what they had been told they should be, they accessed new personal, relational, and creative potential. Noah shared, “So many things that I could not have predicted are now true and vibrant about me, including and founded in my transness.” Particularly, as gender dysphoria lessened and trans joy became more accessible, participants found significant relief from stressors that had previously hindered their growth. This sense of self‐actualization further empowered them to differentiate from parental figures and reclaim agency over their lives.

#### Recognizing systemic harm and cultivating efficacy to resist

Experiencing systemic oppression firsthand while actualizing their TNB identities deepened participants’ critical consciousness of how larger social and contextual structures shape both individual lives and family dynamics. They came to understand that their parental harm was likewise shaped by broader forces such as racism, colonialism, and cis patriarchy. This helped participants reframe the harm they endured, not as a reflection of their own inadequacy, but as a consequence of their parents perpetuating intergenerational trauma shaped by broader structural forces. One participant, Raspberry, reflected, “It wasn't like any one of these people set out to be evil… it is a failure of a much greater scale.” Recognizing these systemic forces helped participants externalize shame and foster empathy for their parents.

Alongside this awareness, participants developed a sense of efficacy and resistance. They began to view themselves as active agents capable of challenging oppression and asserting their truth as forms of active resistance and resilience. Fierce explained that healing allowed them to “stand up and speak my truth and then continue to adapt to what the outcome was.” For TNB participants of color, this resilience was amplified by collective histories of surviving racism and transphobia. BrooklynA emphasized that survival itself became a shared strength.

### Subsequent Strategies for Healing 1: Attempting repair and intergenerational work with families of origin

The consequences of earlier strategies—corrective relating and validation within chosen families, skills and self‐understanding developed in therapy, and resilience and critical consciousness through actualization—created the conditions for participants to attempt repair within their families of origin. With stronger boundaries and a clearer sense of self, participants were able to engage with parents not from a place of vulnerability, but with clarity and agency. Participants shared that being TNB uniquely positioned them to recognize and disrupt patterns of harm. Fly explained, “I think one of the coolest things about transition is it forces you to deal with that stuff…how you feel about gender, how you feel about your parents, how you feel about the way the world looks at you.” Drawing on the consequences of earlier healing strategies, participants sought to name harm; express both pain and empathy; and share insights about gender, race, and coloniality with parental figures. Some participants offered resources such as books or therapy to parents, communicating repair as a simultaneous process of personal repair and intergenerational healing work.

Despite such efforts, the consequences of these repair efforts were mixed. Many repair attempts were met with denial or disengagement, particularly from parents who had perpetrated severe abuse, carried untreated trauma of their own, or resisted engaging in individual healing. Many parents resisted engaging in difficult conversations, withdrawing when harm was named, or alternated between regret and defensiveness. Still, participants emphasized that attempting repair held value even when repair was unsuccessful. The act of repair itself provided closure and liberation, helping participants externalize shame by recognizing the harm as a reflection of their parents’ limitations and unhealed wounds, not their own. This process allowed them to reclaim agency in their relationships and accept their parents’ limited capacity for change, which provided the closure needed to heal on their own terms. In rare cases (two participants), when repair attempts resulted in meaningful accountability, this validated past harm, affirmed the possibility of change, and transformed relationships once characterized by trauma into spaces of safety and closeness.

Not all participants had the opportunity to pursue repair, as circumstances themselves sometimes constrained this strategy. During member checking, one participant shared that they were able to attempt repair with their mother figure but not with their father, who had passed away before the participant came out as trans. They described ongoing grief about the impossibility of repair and its impact on their healing, highlighting the importance of the repair attempt in healing. Another participant chose not to engage in repair with her father, who had been largely absent throughout their life and continued to perpetuate harm through entrenched misogynistic attitudes. For this participant, her father's absence and ongoing harmful behaviors toward women created conditions in which pursuing repair was neither safe nor meaningful. Instead, the participant reclaimed agency by deciding that distancing without repair was most protective and healing.

### Subsequent Strategies for Healing 2: Distancing as an act of self‐preservation

Distancing emerged both as a strategy for healing and a consequence of repair attempts. Initial distancing typically took place after coming out as TNB in response to parental invalidation or hostility. This early distancing often provided participants space to explore their identity on their own terms and form affirming relationships outside the family. As Forest shared, “When I came out as trans, no one supported it…I was kind of able to choose my own family and just be with people that supported me.” Over time, when parents resisted repair or perpetuated harm, participants shifted to more deliberate and permanent forms of distancing, using it as an intentional strategy of self‐preservation.

Participants described a range of distancing practices, from limiting contact to complete disengagement to protect their well‐being. The extent of distancing often depended on parental acceptance of TNB identity: When parents offered at least minimal acceptance, relationships were sometimes maintained, even without full accountability for past harm. For some TNB people of color, distancing was complicated by cultural values of collectivism. In these cases, participants often maintained limited contact, balancing cultural expectations with their own well‐being. Echo shared,

For people of color especially, it can be really difficult to separate family…I never really saw cutting her out of my life completely. But I wanted to make sure…if she's going to be in my life, then I have to make sure that I'm prioritizing myself.

There were significant beneficial consequences from distancing. Participants reported reduced PTSD symptoms, improved emotional regulation, and a stronger sense of self. Thunder reflected, “The healing became real…it was less pain. And I just felt better about myself overall. It gives me the ability to breathe more often.” Distancing also helped participants build efficacy in setting firmer boundaries in other relationships.

Distancing from parents brought more relief than grief. It offered a release from the ongoing harm and emotional burden of attempting to repair unchanging relationships. When grief or guilt surfaced, participants processed these emotions without being drawn back into harmful dynamics. As Thunder shared, “Every now and then, I'll think about things I used to love about [my father]. And it really does hurt. But I am never going back there. I never will.” Although grief and relief often coexisted, relief ultimately carried more weight.

### Subsequent Strategies for Healing 3: Strengthening a safer TNB home and holding space for the next generation of healing

Ultimately, participants brought the wisdom and strength gained from their healing journeys back into the queer and TNB communities that had sustained participants’ recovery. In doing so, they contributed to a generative cycle of collective care—supporting the next generation of survivors navigating similar parental trauma. BrooklynA described supporting younger queer and trans people who had endured parental harm as both an act of service and a continuation of their own healing:

I want to help young queer and trans folks who may have had a mother like I did. … Doing this work has been a huge part of my own trauma, my own resilience, my own joy. It's been a blessing for me.

This return to TNB community was not only an outcome but also an active, ongoing strategy of healing. By sustaining collective healing spaces and offering care to others, participants reinforced the very communities that had supported their own healing. In this way, healing became cyclical: individual healing transforming into collective healing.

## DISCUSSION

The present study developed a theoretical framework explaining how TNB individuals heal from relational trauma with parental figures. This model conceptualizes healing as an iterative process of conditions, strategies, and consequences in which TNB identity and experience become central pathways of recovery. It demonstrates that TNB identity actively structures the process of healing from the relational trauma—from conditions that foster self‐recognition and awareness to strategies of relational and intrapersonal healing and to consequences that sustain both personal and collective healing (see Figure 1).

Contrary to narratives framing transness primarily as a source of relational trauma or a risk factor for traumatic stress, our findings reveal that TNB identity and related experiences facilitate access to the very conditions necessary for healing from parental relational trauma. Participants emphasized that their transness did not *cause* parental harm but created conditions to *reveal* preexisting patterns of maltreatment. This finding may help explain higher reported rates of relational trauma in TNB communities (Thoma et al., 2021); prevalence rates may reflect an increased capacity to recognize and name traumatic experiences. When parents redirected abuse toward participants’ gender identity, it amplified and clarified longstanding harm in ways that other forms of rejection had not. This recognition served as a critical condition in breaking cycles of relational trauma and establishing relational safety elsewhere, a task many trauma survivors find challenging (Herman, 2015).

Connecting with and actualizing one's TNB identity also fostered self‐attunement, decreased dissociation, and a clearer self‐concept, all of which are vital for resilience after parental harm (Fares‐Otero et al., 2025; Stige et al., 2013). By constructing self‐definitions that diverged from parental projections, participants enacted a key process of recovery identified in relational trauma treatment (Courtois & Ford, 2012; Fishbane, 2019). Furthermore, trusting the authenticity of their own experiences despite external invalidation cultivated self‐trust and assertiveness, which are core elements of healing from chronic invalidation and shame (Stige et al., 2013).

Corrective relational experiences are central to healing from traumatic parental relationships (McCormack & Katalinic, 2016), yet prolonged relational trauma often leads survivors to repeat unhealthy relational patterns and experience revictimization (Courtois & Ford, 2012). For TNB people, parental rejection that occurred alongside societal oppression established conditions for participants to create chosen families that fostered relational safety and helped integrate shared traumatic experiences. Within these chosen queer and TNB families, participants experienced corrective experiences of unconditional care, the ability to safely navigate conflicts, and mutually respectful boundaries, all of which are known sources of healing from relational trauma (Fares‐Otero et al., 2025; McCormack & Katalinic, 2016).

A key nuance of this strategy was the role of shared intersecting identities (e.g., TNB, queer, race, socioeconomic status) in cultivating relational safety. For participants of color, shared racial identities within queer and TNB chosen families were crucial for accessing relational safety. This need stemmed from experiences of White supremacy, microaggressions, and a lack of confidence that White TNB and queer individuals could fully understand the oppression that TNB people of color faced. These findings extend prior understanding regarding the importance of intersectional community in fostering resilience from trauma among TNB people of color (Singh & McKleroy, 2011).

Validation of traumatic experiences was a crucial process in participants’ healing. Beyond developing self‐validation through increased self‐trust, participants found that shared experiences within TNB and queer communities facilitated coprocessing and external validation of trauma. This communal meaning‐making supported narrative integration, a core process of trauma recovery (Grossman et al., 2017; Herman, 2015).

Therapy also emerged as a vital context for validation, offering structured opportunities to understand the impact of trauma and reduce shame. Consistent with research on trauma‐focused psychotherapy (Lewis et al., 2020), participants described therapy as an important site of healing. Many sought therapy to address the intersecting challenges of relational trauma and minority stress, often with the encouragement of their chosen families.

For most TNB survivors in this sample, distancing from parents was an essential strategy for healing. Despite this need, participants were not without empathy for their parents. Through actualizing minoritized identities and connecting with chosen families, participants developed critical consciousness that allowed them to contextualize parental behavior within broader systems of oppression and intergenerational trauma. This empathy enabled many participants to engage in intergenerational work—steps their cisgender siblings often could not take. Whether or not a repair occurred, this strategy helped participants externalize shame, recognizing harm as stemming from their parents’ unhealed wounds rather than their own. Participants also came to accept their limited control over the family system and established effective boundaries, consistent with therapeutic models of recovery (Fishbane, 2019). Importantly, our findings demonstrate that repairing the parental relationship is not a prerequisite for recovery. The repair attempt and closure served as a healing process in itself, allowing participants to move forward.

Participants who experienced meaningful recovery frequently extended care to others, creating cycles of collective healing. By sharing their stories, offering mentorship, and building supportive spaces, participants contributed to others’ recovery while deepening their own. This reciprocal process aligns with Herman's (2015) final stage of trauma recovery (i.e., reconnection and integration) and with research identifying community mentorship as a resilience factor among TNB individuals (Matsuno & Israel, 2018).

Several limitations of the present study merit discussion. Our recruitment strategy likely yielded a sample that was particularly oriented toward community connection, which may not reflect the experiences of TNB individuals for whom such networks are less central or accessible. Future research should recruit participants from a wider range of settings, such as primary care clinics that provide hormone therapy or general mental health practices, to examine whether differing levels of community connection influence healing processes. That said, centrality of community to TNB well‐being and resilience is well‐documented in prior research (Matsuno & Israel, 2018).

Although demographic diversity was achieved, all participants were TNB adults residing in the United States. Given that intergenerational histories, familial norms, and access to TNB communities and gender‐affirming care can vary significantly globally, cross‐cultural research is needed to explore how these healing processes unfold in different sociopolitical contexts. Additionally, with only one participant over 60 years of age, the experiences of older TNB adults—who came of age prior to increased community visibility—remain underrepresented. Future studies should examine how historical context, age‐related factors, and intergenerational dynamics shape healing among TNB elders.

Our findings partially support the applicability of existing models of therapy for relational trauma while underscoring the need for TNB‐specific adaptations. In particular, therapists should promote self‐concept clarity and differentiation through the affirmation and actualization of TNB identity. Participant experiences further suggest that facilitating access to supportive communities and gender‐affirming medical care can help clients develop critical awareness of how intergenerational trauma and systemic oppression (e.g., transphobia, racism) shape family systems and personal experiences. Participants also emphasized the centrality of chosen family as sources of affirmation, care, and belonging, highlighting the importance of therapeutic models that move beyond the nuclear family paradigm to support chosen kinship networks as primary sites of healing. Recent efforts to expand family therapy models to include chosen kin represent a promising direction for this work (Jenicek & MacIntosh, 2022). Accordingly, fostering corrective relational experiences within shared, intersecting communities is critical. From a clinical perspective, these findings suggest that interventions intentionally designed to strengthen intersectional community connection and gender affirmation may be critical for supporting recovery from parental relational harm.

An important clinical implication of this study is that distancing from biological family can be a legitimate—and sometimes essential—pathway to healing. Although family therapy literature often prioritizes reconciliation and reunification (Hughes, 2007), our findings indicate that these processes are not always feasible or desirable. Therapists must be open to the value of temporary or permanent distancing from parents and support clients establish and reevaluate boundaries while acknowledging the complex long‐term emotional experiences (e.g., grief and relief) that follow estrangement.

During member checking, participants reflected that research participation itself was healing, as it allowed them to construct narratives framing their transness as an asset. This highlights the value of narrative and trauma‐focused therapies that integrate not only experiences of trauma and transnegativity (e.g., Lange, 2020) but also trans‐related strengths. We recommend that therapists intentionally attune to and highlight how transness facilitates safety, healing, and growth. Empowering TNB individuals to internalize their transness and intersectional identities as sources of strength holds immense healing potential, especially amid sociopolitical hostility that pathologizes TNB identities (Ashley, 2023).

This study's central finding that TNB identity is a source of relational and intergenerational healing, rather than solely a source of disruption or vulnerability, has direct implications for advocacy and community organizing. Promoting this empirically grounded, strength‐based perspective is a vital tool to combat stigma, foster identity pride, and challenge hostile sociopolitical narratives. Because gender‐affirmation, competent therapy, and community connection are primary pathways to healing, advocacy efforts must focus on dismantling barriers to these resources. This includes expanding access to gender‐affirming medical care, making therapy affordable and accessible, and ensuring the provision of comprehensive cultural competence training for clinicians. Finally, advocacy should center on creating and protecting community spaces that offer TNB individuals the unconditional acceptance and relational safety necessary for recovery and well‐being.

## AUTHOR NOTE

This project was supported by the American Psychological Foundation (Roy Scrivner Memorial Research Grant) and the University of Wisconsin–Madison (Baldwin Seed Grant).

## OPEN PRACTICES STATEMENT

Given the qualitative design and the sensitive nature of the data, the data have not been made available on a permanent third‐party archive in order to protect participant confidentiality, in accordance with Institutional Review Board requirements. The semi‐structured interview protocol is included in the Supplementary Materials. Requests for additional information may be directed to the lead author at jlee994@wisc.edu.

## AUTHOR CONTRIBUTIONS

**Joonwoo Lee**: conceptualization, methodology, data curation, project administration, formal analysis, investigation, visualization, funding acquisition, writing ‐ original draft, writing ‐ review and editing. **Sebastian Barr**: conceptualization, methodology, data curation, formal analysis, investigation, writing ‐ original draft, writing ‐ review and editing. **Alberta M. Gloria**: writing ‐ review and editing, supervision. **Tonya J. Roberts**: supervision, methodology, writing ‐ review and editing. **Stephanie L. Budge**: supervision, writing ‐ review and editing.

## Supporting information



Supplementary Materials
