# Supplementary material for: Transness is our salve: How trans identity facilitates healing from relational trauma with parental figures
Source: J Trauma Stress. 2026 Jan 28;39(4):616–28. doi: 10.1002/jts.70044 (PMC13436463; doi:10.1002/jts.70044)
Supplement: Supplementary file 1 [file JTS-39-616-s001.docx]

**Semi-Structured Interview Protocol**

This document outlines the semi-structured interview protocol used in our study. Consistent with Constructivist Grounded Theory (CGT), the protocol evolved from a broad, exploratory phase (Phase 1: Purposive Sampling) to a more focused, theoretically driven phase (Phase 2: Theoretical Sampling) as key categories emerged from the data. The questions below served as a flexible guide rather than a fixed script.

**Phase 1: Purposive Sampling Protocol**

*(Used for initial interviews to broadly explore the phenomena of relational trauma and healing)*

**I. Pre-Interview**

- Welcome, Introduction of the Study, Informed Consent
- **Defining Terms and Facilitating Safety:**
  - “Throughout this interview, I will ask questions about your relationship with parental figures, specifically experiences of trauma involving parental figures. Who would you include in your definition of ‘parents’?”
    - *Follow-up: Where appropriate, inquire about additional parental figures (e.g., stepparents, guardians, foster parents).*
  - “How would you like me to refer to them during our conversation?” (e.g., mom, dad, grandmother, or any preferred term)
  - “I understand that what we talk about today can be stressful and hard to talk about, and it’s important for us that this interview is not only helpful to us as we understand your experience but for you to process and explore your life story. Please let me know if you’d like to pause, skip any question, or take a break. There are no right or wrong answers. If an experience mattered to you, it matters to us.”

**II. Main Interview Questions**

1. “For many people, relationships with parents change over time. How did your relationship with your [preferred term(s)] change for you?”
2. “What events or circumstances with your [preferred term(s)] did you experience as traumatic? Feel free to share as much as you feel comfortable.”
3. “How do you think your relationship with your [preferred term(s)] has shaped who you are today?”
4. “How did your relationship with your [preferred term(s)] affect how you relate and connect with other people?”
5. “How has your relationship with your [preferred term(s)] affected your relationship with your trans identity?”
6. “How have you been healing from wounds/distress from relationship trauma with [preferred words for parent(s)]?”
   - *Follow-ups:* What has helped your healing? Who has supported your healing? How has this healing impacted you?
7. **Exploring Common Impacts of Trauma:**
   - “I’d like to ask some questions about common impacts of relational trauma. For context, ‘parental trauma’ here refers to repeated experiences of emotional or physical abuse or neglect from parents that have had lasting negative impacts on your sense of self, mental and physical health, and relationships with others.”
   - “These questions are not meant to diagnose but to help give language to your experiences. Trauma impacts everyone differently, so some questions may feel more or less relevant to you. Please share what feels true for you.”
   - Then, we reviewed the items from the PTSD subscales of the International Trauma Questionnaire (Cloitre et al., 2018) and asked participants to discuss how frequently they experienced each symptom and how relevant it was to them.

**III. Post-Interview & Debriefing**

1. **Debrief Script:**
   - “I’d like to explain why I asked some of the questions that I did today. I will take a couple of minutes to walk you through what we call a debrief. After that, I’m going to ask you for how today felt & feedback for our interview. I will send you the debrief guide by email.”
   - *(Review Debrief Guide)*
2. **Participant Feedback:**
   - “How was it to share your story with me today?”
   - *Follow-ups:*
     1. What helped you feel safe or comfortable?
     2. Were there moments you felt uncomfortable?
     3. If you were interviewing someone about this, what might you do differently?
     4. Is there anything you’d like us to do differently in future interviews?

**Phase 2: Theoretical Sampling Protocol**

*(Used for Phase 2 interviews to clarify, challenge, and elaborate on emerging theoretical categories such as “intergenerational repair,” “chosen family,” “therapy,” and “distancing.”)*

**I. Pre-Interview Questions**

*(This section remained consistent with Phase 1 to ensure rapport and participant safety, with one additional question to contextualize the discussion.)*

- “I recall from your initial survey that you mentioned experiencing some challenges in your relationship with your parental figures, which have deeply affected you. If you feel comfortable, could you please share a bit more about what aspects of your relationship with your parent felt particularly traumatic for you? This does not need to include details.”

**II. Focused Theoretical Questions**

*In this phase, questions shifted from broad exploration to targeted inquiry about specific concepts identified in earlier interviews. Questions were framed to elicit richer detail.*

- “How have you been healing from wounds/distress from relationship trauma with [preferred words for parent(s)]?”
- **Probes on Emerging Categories:**
  - **Role of Chosen Family**
    - “On the survey, you mentioned connection with chosen family as a key part of your healing. Can you tell me about the role that community or chosen family has played in your recovery from parental trauma?”
  - **Intergenerational Repair & Distancing**
    - “On the survey, you mentioned [participant’s experience with repair or distancing]. Could you share the story of how you came to that place with your parent(s)?”
      - If repair was attempted:
        - “What did that repair process look like? What made it possible–or not possible? How has that affected your healing?
      - If distancing was attempted:
  - “What led you to create distance? What made it possible–or not possible? How has that affected your healing?
  - **Cultural Context**
    - How do you think your racial/ethnic/cultural identity background(s) played a role in your healing?”
  - **Impact of Healing on Self and Relationships**
    - “How did the healing/repair work with your parents impact/change...”
      - “...how you relate to others?”
      - “...how you relate to self?”
      - “...how you relate to your trans identity?”
      - “...how you relate to the TNB community?”
- **Exploring Changes in Trauma Impacts**
  - “I am going to ask some questions about your experiences of other common impacts of relational trauma... As we go through these questions, let me know how you have experienced or did not experience changes in these symptoms” *(Use the same Table from Phase 1)*

**III. Post-Interview & Debriefing** *(This section remained consistent with Phase 1).*
